# Predicting myopic changes in children wearing glasses using the Plusoptix photoscreener

**DOI:** 10.1007/s10792-024-02954-9

**Published:** 2024-02-16

**Authors:** Sandra Guimaraes, Maria João Vieira, José Miguel Vilas Boas

**Affiliations:** 1 FP-I3ID (Instituto de Investigação, Inovação e Desenvolvimento da Universidade Fernando Pessoa), Porto, Portugal; 2 HE-UFP (Hospital-Escola da Universidade Fernando Pessoa), Av. Fernando Pessoa 150, São Cosme, 4420-096 Portugal; 3 FCS-UFP (Faculdade de Ciências da Saúde da da Universidade Fernando Pessoa), Porto, Portugal; 4 Escola de Medicina da Universidade do Minho, Braga, Portugal

**Keywords:** Plusoptix, Ophthalmological screening, Pediatric ophthalmology, Myopia, Myopia progression

## Abstract

**Introduction:**

With high increase in myopia prevalence, we aimed to assess whether Plusoptix_A09 can be used in myopic children over spectacles to predict visual acuity (VA) and myopic refraction changes.

**Methods:**

Myopic children underwent a complete ophthalmological examination. Plusoptix_A09 was performed over spectacles. VA changes, refraction changes and time since previous glasses prescription, were determined. Age, current or past history of amblyopia, presence of strabismus and self-perception of VA changes were registered.

**Results:**

In total, 199 patients were included. Spherical power (SP) and spherical equivalent (SE) measured by Plusoptix_A09 over spectacles predicted both VA changes (*p* < 0.001) and refraction changes (*p* < 0.001). Values of SP < − 0.06D or SE < − 0.22D indicated a VA decrease (AUC > 0.9, *p* < 0.01) for sensitivity and specificity of 85.1%, 82.1% and 82.6%, 83.3%, respectively. Age and ophthalmological comorbidities did not influence Plusoptix_A09 measurements (*p* > 0.05). Plusoptix_A09 over spectacles was a stronger predictor of VA changes when compared to children's self-perception, either in 4–9-year-old patients (*p* < 0.001 versus *p* = 0.628) and in 10–18-year-old children (OR <  = 0.066 versus OR = 0.190). A decrease in SP and SE of − 0.10D in Plusoptix_A09 predicted a myopia progression of − 0.04D and − 0.05D, respectively.

**Conclusion/Relevance:**

This study unveiled new features for the Plusoptix, a worldwide available photoscreener used in amblyopia screening. When Plusoptix is performed in children with their glasses on, it can rapidly predict myopia progression. For each decrease of − 0.10D in Plusoptix, a myopia progression of -0.05D is expected. Moreover, Plusoptix is more reliable than children's self-perception of visual acuity changes, making it a useful tool either in primary care or ophthalmology practice

## Introduction

Myopia is continuously progressive affecting the refraction needed to reach the best-corrected visual acuity (BCVA) [1]. It is clear that myopic children who require refractive correction with glasses should be frequently reevaluated to assess myopia and VA progression [2].

Plusoptix_A09 photorefractor is a noninvasive, handheld and easy-to-use device that performs binocular real-time videoretinoscopy of reflected infrared light in order to rapidly identify refractive errors (RE) in children [3]. It measures refractive data, pupil size, interpupillary distance, asymmetry of the corneal reflexes and gaze deviation without the need to perform cycloplegia [4, 5]. During examination, Plusoptix is placed approximately 1 m from the patient and then the device produces noises and flashing lights to fix child’s gaze and the respective measurements are obtained [4]. During its standard use, children are not using any type of refractive correction [4]. The effectiveness of noncycloplegic autorefraction with Plusoptix in detecting myopia, astigmatism and anisometropia has been extensively studied, and it is reliable when compared to cycloplegic autorefraction and retinoscopy (it has lower acuity in the detection of hyperopia, though) [6]. Moreover, it has also been demonstrated that photoscreening with Plusoptix is similar to traditional chart-based screening in the detection of VA impairment and the need for glasses or a new intervention in school-age children [7].

Although Plusoptix has proven to be useful in detecting both RE and VA impairment when performed in children without correction, to our knowledge, there are no studies on the performance of Plusoptix over spectacles and its potentials as a follow-up tool in myopic children.

The purpose of this study is to evaluate the performance of PLUSOPTIX_A09 in predicting VA and myopia changes in children over their spectacles. Furthermore, we assessed whether individual and eye-related factors influence the performance of PLUSOPTIX_A09: In addition to age, which has shown to have an impact in VA progression [8], the presence of past and current history of amblyopia and the time since last glasses prescription were also included for analysis. Although the use of the photoscreener without correction is limited in children with strabismus [9], some strabismus can be totally or partially controlled with the glasses on [10], making it possible to perform a binocular measurement with the PLUSOPTIX_A09. To evaluate its influence in the PLUSOPTIX_A09 measurements, the presence of strabismus was also considered for analysis.

## Materials and methods

All myopic children wearing glasses, aged 4–18 years old, that attended an ophthalmology consultation between February 2020 and May 2021, in whom PLUSOPTIX_A09 was performed over their spectacles, were included. PLUSOPTIX_A09 measurements were always taken with the children wearing their current spectacles (there are no Plusoptix measurements without glasses in this work), and for that reason the expression “PLUSOPTIX_A09” in this study always refers to measurements done over current spectacles. Measurements using the Plusoptix_A09 were taken with the child wearing glasses, at the distance similar to that used for children without glasses. These measurements were taken binocularly, focusing solely on Plusoptix_A09. In cases where reflections occurred, adjustments were made by slightly rotating the child's head to ensure accurate measurements.

Plusoptix is developed and manufactured by Plusoptix GmbH, in Nuremberg, Germany. All Plusoptix devices use the measuring principle of the transillumination test; however, they avoid glare by using infrared light. The device records camera images of the illuminated pupils and measures the refraction.

Myopia or myopic astigmatism was considered present when spherical equivalent (SE) <  = -0.50D and values of the spherical power (SP) <  = zero [11]. All consecutive participants underwent complete ophthalmological examination performed by an experienced pediatric ophthalmologist.

Exclusion criteria were: Whenever binocular evaluation with PLUSOPTIX_A09 over spectacles was not possible (large-angle strabismus not corrected with spectacles, ptosis, corneal opacities and poor ocular fixation); RE exceeding the range of PLUSOPTIX_A09 (− 7.00D to + 5.00D); noncooperative children; inadequate clinical records.

Information collected from the electronic clinical database was: Age; Gender; Presence of current and past history of amblyopia; Presence and type of strabismus; Prescription of current glasses; Self-perception of VA changes (later described in this section); VA with correction (VAwc), which refers to patient’s VA with current glasses; Time since current glasses prescription; The average of two binocular measurements with the PLUSOPTIX_A09 (of SP, cylinder power (CP) and SE); BCVA that indicates the best-corrected visual acuity that could be achieved with a new refraction; Refractive errors obtained from subjective refraction and cycloplegic autorefraction (after instillation of 3 drops of cyclopentolate 1.0% in each eye, with 10-min interval, followed by autorefractometer, either KOWA KW-2000® or HUVITZ HRK-8000A®, measurement after 40 min).

VA was evaluated monocularly either with single surrounded thumbling E or numbers chart displayed in MediWorks C901® video system. VA was recorded in decimal notation and computed into LogMAR VAwc and LogMAR BCVA [12]. [LogMAR BCVA–LogMAR VAwc] indicates the lines of VA the patient is away from reaching the best VA possible. Since a VA test–retest variability within 0.1 LogMAR can occur [13], a new categorical dichotomic variable was established—VA status: stable VA: 0 ≤ [LogMAR BCVA–LogMAR VAwc] ≤ 0.1 LogMAR versus decreased VA: [LogMAR BCVA–LogMAR VAwc] > 0.1LogMAR.

When new refraction was different from current glasses, a new prescription of glasses was obtained, considering cycloplegic autorefraction and subjective refraction. To quantify RE progression, a new continuous variable was created: refraction changes, which corresponded to the difference between new refraction (new glasses prescription) and current refraction (previous glasses prescription) for the SP, CP and SE.

To evaluate self-perception of VA changes, children were asked “Do you think you still have a good vision with your glasses?” and answers of “yes” or “no” were registered.

In this work, reports refer to the analysis of the right eye (or left eye when the right eye was emmetrope) [14].

This project was approved by the local ethics committee.

### Statistical analysis

To compare PLUSOPTIX_A09 measurements, age and time since previous glasses prescription between groups of VA status independent samples t test or Mann–Whitney U test (nonparametric alternative) were used; effect size: Cohen’s* d* [small 0.2, medium 0.5, large 0.8 [15]]. Pearson’s Chi-square test was performed to assess differences in the presence of ophthalmological abnormalities between groups of VA status; effect size: phi/crammer’s V [small 0.1, medium 0.3, large 0.5 [15]]. A univariate logistic regression model was estimated to assess whether PLUSOPTIX_A09 predicted VA changes. A multivariate logistic regression analysis was used to study the influence of age, time since previous prescription and the presence of ophthalmological abnormalities in the predictive power of PLUSOPTIX_A09. Since no evidence or guidelines concerning the performance of Plusoptix in children with glasses have yet been published, a receiver operating characteristic (ROC) curve analysis was performed in order to calculate cutoff points for PLUSOPTIX_A09 measurements over spectacles to identify children with stable and decreased VA [16]. To evaluate whether PLUSOPTIX_A09 were predictors of refraction changes, the following criteria were used: Pearson’s correlation coefficient and Spearman's rank correlation coefficient were calculated to assess the correlation between both measurements (when significant, Pearson’s coefficient was reported [small 0.1, medium 0.3, large 0.5 [15]); then, the PLUSOPTIX_A09 measurements that significantly correlated with refraction changes were included in a simple linear regression to assess whether PLUSOPTIX_A09 predicted refraction changes and to determine its predictive power. Pearson’s Chi-square test of independence was used to study the association between children’s self-perception of VA changes and the presence of VA changes in at least one eye detected during medical examination; effect size was phi [small 0.1, medium 0.3, large 0.5 [15]]. For this analysis, subjects were divided into two age groups: the 4–9 year old and the 10–18 year old. Then, to compare the predictive powers of the PLUSOPTIX_A09 measurements with self-perception of VA changes, the univariate logistic regression model was used to calculate the odds ratio (OR). The statistical analyses were performed using the Statistical Package for the Social Sciences (IBM SPSS®), version 27.0. Two-sided *p*-values < 0.05 with confidence interval (CI) of 95% were considered statistically significant.

## Results

A total of 199 participants were included; 109 females (54.8%); mean age of 10.8 years old (SD = 3.2). Nineteen patients (9.5%) had past history of amblyopia, and 2 children (1%) had current history of amblyopia. Strabismus was present in 10 subjects (5%): 3 (1.5%) with esotropia and 7 (3.5%) with exotropia. The median time since previous prescription was 13.0 months (IR = 10.0). Of the total sample, 121 (60.8%) children had stable VA and 78 (39.2%) had decreased VA with current glasses.

### Prediction of VA status (stable versus decreased VA)

Regarding VA status, PLUSOPTIX_A09 measurements were significantly different for SP and SE [+ 0.35 ± 0.53D versus − 0.98 ± 0.92D, *t*(110) = 11.58, *p* < 0.001, *d* = 1.87; and + 0.13 ± 0.51D versus − 1.21 ± 0.91D, *t*(108) = 11.80, *p* < 0.001, *d* = 1.92, respectively], but not for CP [*p* = 0.980].

As shown in Table 1, both the SP and SE measurements with PLUSOPTIX_A09 were significant independent predictors of VA changes and, in the adjusted model, their ORs even improved. Time since previous prescription was a weaker predictor than PLUSOPTIX_A09 measurements. Age, history of strabismus and amblyopia were nonsignificant in predicting VA status in the multivariate analysis (Table 1).

**Table 1:** Univariate and multivariate logistic regression for Plusoptix_A09 SP and SE in children with myopia or myopic astigmatism

	Variable	*B*	SE	Wald	*p*-value	OR	95% CI		*R*^2^
							Lower	Upper	
SP Univariate	Plusoptix_A09 SP	− 2.792	0.406	47.210	< 0.001*	0.061	0.028	0.136	0.611
SP Multivariate	Plusoptix_A09 SP	− 3.050	0.475	41.218	< 0.001*	0.047	0.019	0.120	0.676
	Age	− 0.138	0.081	2.937	0.087	0.871	0.744	1.020	
	Past amblyopia	− 0.887	0.820	1.170	0.279	0.412	0.083	2.055	
	Strabismus	0.975	0.552	3.117	0.077	2.651	0.898	7.822	
	Time since previous glasses prescription	− 0.066	0.031	4.424	0.035*	0.936	0.881	0.996	
SE Univariate	Plusoptix_A09 SE	− 2.798	0.405	47.844	< .001*	0.061	0.028	0.135	0.617
SE Multivariate	Plusoptix_A09 SE	− 3.033	0.470	41.578	< .001*	0.048	0.019	0.121	0.679
	Age	− 0.121	0.081	2.264	0.132	0.886	0.757	1.037	
	Past amblyopia	− 0.841	0.852	0.976	0.323	0.431	0.081	2.288	
	Strabismus	0.830	0.533	2.427	0.119	2.294	0.807	6.517	
	Time since previous glasses prescription	− 0.071	0.032	4.954	0.026*	0.932	0.875	0.992	

Plusoptix_A09 measurements were always performed with children wearing their glasses

To avoid multicollinearity, current amblyopia was not considered for this analysis

^*^Significant *p*-value (< 0.05). SP: spherical power, SE: standard error, OR: odds ratio, CI: confidence interval, *R*^2^: Nagelkerke's R squared

**Fig. 1:**
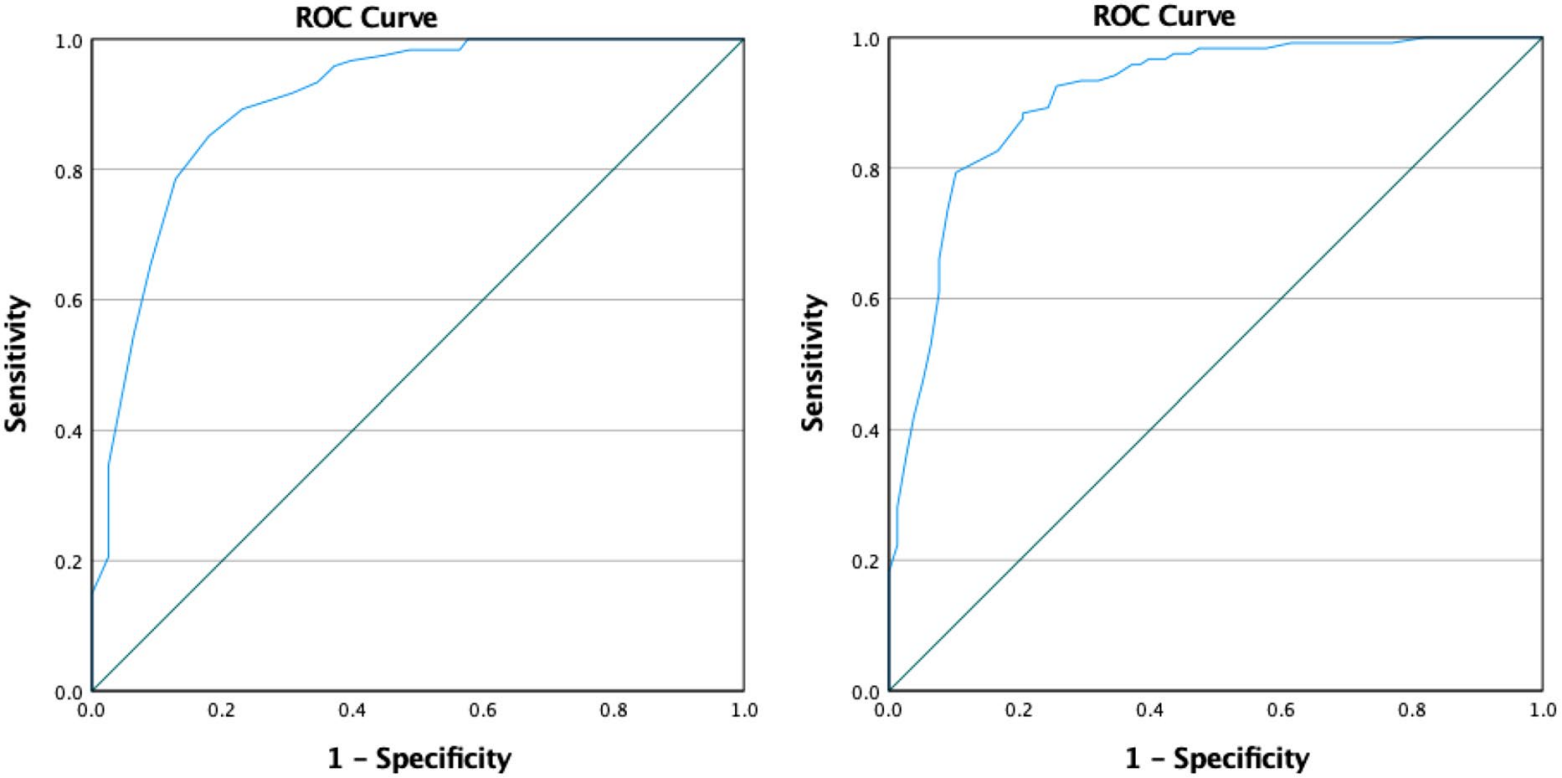
ROC curve of the performance of Plusopotix_A09 SP (left graph) and SE (right graph) in detecting visual acuity changes in children with myopia or myopic astigmatism. A value of SP or SE worse (more negative) than − 0.06D or − 0.22D, respectively, indicates a decreased VA with current glasses (sensitivity and specificity of 85.1% and 82.1% for SP and 82.6% and 83.3% for SE); AUC for SP = 0.904, 95%CI = 0.860–0.949, *p* < 0.01. AUC for SE = 0.910, 95%CI = 0.867–0.953, *p* < 0.01. Plusoptix_A09 measurements were taken over spectacles. Significant *p*-value (< 0.05). SP, spherical power; SE, spherical equivalent; AUC, area under the curve; CI, confidence interval.

The ROC curve analysis showed significant predictive power of both PLUSOPTIX_A09 measurements in detecting VA changes (AUC > 0.9, *p* < 0.01). These results suggest that a value of SP or SE measured by PLUSOPTIX_A09 worse (more negative) than -0.06D or -0.22D, respectively, indicated that children with myopia or myopic astigmatism had decreased VA with current glasses, with a sensitivity and specificity of 85.1% and 82.1% for SP and 82.6% and 83.3% for SE (Fig. 1).

### Prediction of refraction changes

**Fig. 2:**
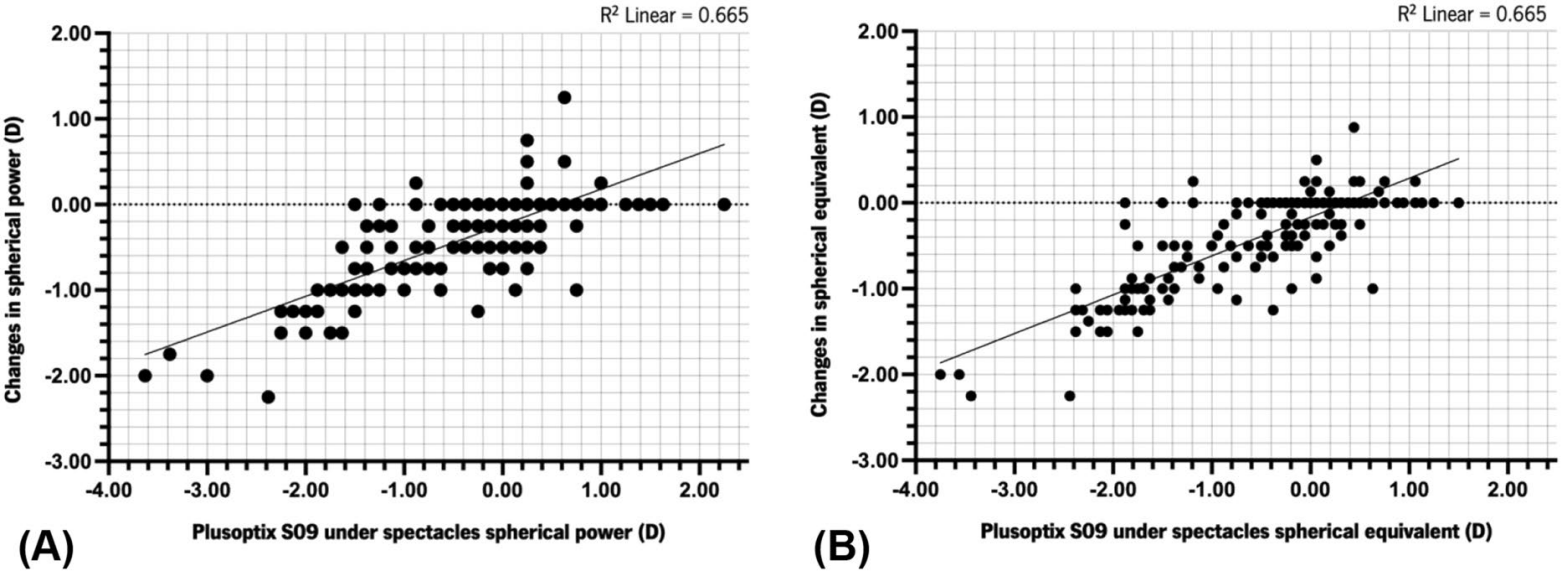
Scatter plots illustrating the linear relationship between Plusoptix_A09 measurements over spectacles and changes in the spherical power (**A**) and in the spherical equivalent (**B**) detected during ophthalmological examination in children with myopia or myopic astigmatism. Plusoptix_A09 measurements were taken over spectacles. D, diopter. R^2^, R squared of linear regression

SP and SE measured with the PLUSOPTIX_A09 explained a significant amount of variance in refraction changes [SP: *F*(1,197) = 316.64, *p* < 0.001,* R*^*2*^ = 0.665; SE: *F*(1,197) = 390.21, *p* < 0.001, *R*^*2*^ = 0.665]. The regression coefficients indicated that a decrease in SP and SE of − 0.10D estimated by the PLUSOPTIX_A09 over spectacles predicted a myopia progression (a more negative SP and SE) of − 0.04D and -0.05D, respectively (SP: *B* = 0.418, 95%CI = 0.372–0.464; SE: *B* = 0.453, 95%CI = 0.408–0.498). Figure 2 illustrates the linear regression model between PLUSOPTIX_A09 measurements over spectacles and changes in the SP and SE detected during ophthalmological examination. Children with stable and decreased VA had similar CP measurements with the PS09 over spectacles [− 0.38 ± 0.31D vs. − 0.38 ± 0.38D, *U* = 4729.00, *p* = 0.980, *r* = 0.00].

### Children’s self-perception of VA changes

Self-perception of VA changes was assessed in 72 consecutive myopic patients. From those, 27 (37.5%) were 4–9 years old and 45 (62.5%) were 10–18 years old.

In the age group 4–9 years old there was no significant association between self-perception of VA changes and detected VA changes in at least one eye (*p* = 0.628 by Fisher’s exact test). However, results were considerably different in the age group 10–18 years old, where a significant association with a moderate effect size was found [*Χ*^*2*^(1, N = 72) = 5.993; *p* = 0.014; *ɸ* = − 0.365].

To compare whether PLUSOPTIX_A09 measurements or self-perception of VA changes are stronger predictors of VA changes, the univariate logistic regression model was used to calculate the ORs. For this analysis only 10–18-year-old children were considered, since prior analysis only found significant results for this age group. All variables were significant predictors of VA changes, although PLUSOPTIX_A09 measurements expressed stronger ORs when compared to children’s self-perception (Table 2).

**Table 2:** Logistic regression for SP and SE measured by Plusoptix_A09 over spectacles and for children’s self-perception of visual acuity changes, in children with myopia or myopic astigmatism included in the age group 10–18 years old

	Variable	*B*	SE	Wald	*p*-value	OR	95% CI		*R*^2^
							Lower	Upper	
Univariate	Plusoptix_A09 SP	− 2.819	0.524	28.931	< 0.001*	0.060	0.021	0.167	0.552
Univariate	Plusoptix_A09 SE	− 2.715	0.497	29.813	< 0.001*	0.066	0.025	0.175	0.558
Univariate	Self-perception of VA changes 10-18y (at least one eye)	− 1.658	0.705	5.526	0.019*	0.190	0.048	0.759	0.169

Plusoptix_A09 measurements were taken over spectacles

^*^ Significant *p*-value (< 0.05). SP: spherical power, SE: spherical equivalent, SE: standard error, OR: odds ratio, CI: confidence interval; *R*^2^: Nagelkerke's R squared

## Discussion

Refractive eye problems in children have become a global public health challenge with myopia alone being acknowledge as a growing epidemic [17, 18]. Therefore, reliable and affordable tools for monitoring myopia in children need to be developed [19].

Plusoptix is a worldwide available photoscreener, used by many pediatricians, primary care physicians and ophthalmologists [20], but has not yet been studied in children over their glasses. Thus, this is the first study, to our knowledge, to explore the potentials of the Plusoptix in children wearing spectacles. Our aim was to find new features for this extensively common photoscreener that could help ameliorate the increasing demand of specialized eye care [21].

### VA changes

Based on our results, performing Plusoptix in myopic children over spectacles is an effective and easy-to-use method to rapidly identify a decrease in VA. Our results revealed that both SP and SE measured by PLUSOPTIX_A09 over spectacles are able to independently predict a decrease in VA with current glasses. As SP and SE get more negative, the odds of VA worsening significantly increase (OR = 0.061 for both SP and SE). Our ROC curve analysis of decreased versus stable VA suggests that more negative (more myopic) values of SP or SE indicate progression of myopia: For cutoffs of SP < − 0.06D and SE < − 0.22D, PLUSOPTIX_A09 over spectacles detects myopia progression with a sensitivity of 85.1% and 82.6% and a specificity of 82.1% and 83.3%, respectively. On the other hand, CP measured by PLUSOPTIX_A09 over spectacles does not predict a decrease in VA since CP is more stable over time.

As Plusoptix is an user-friendly device, it can be easily performed both at specialized and primary care levels [20, 22]. At a specialized level, the pediatric ophthalmologist can use the Plusoptix over spectacles as an initial screening tool to rapidly assess whether a myopic child has decreased their VA. At a primary care level, Plusoptix seems to be even more useful, especially because vision screening in this setting is time-consuming and has low reimbursement, besides many physicians report to be not adequately trained to perform VA testing [23]. By routinely performing Plusoptix in children over spectacles and considering the calculated cutoff points, physicians can easily and rapidly identify children who may require referral to update glasses prescription.

As expected, children with stable VA were older, since myopia gradually stabilizes with age [24]. Furthermore, children with stable VA also experienced longer time since previous glasses prescription. These patients might have slower progressing myopia [25], with a less often need to update their prescription. When assessing the influence of ophthalmological comorbidities, PLUSOPTIX_A09 remains a strong predictor of VA progression even in children with current or past history of amblyopia or with strabismus (OR = 0.047 for SP and OR = 0.048 for SE, in the adjusted model). This finding looks especially interesting, because although binocular measurements with Plusoptix cannot be performed without glasses in children with significant strabismus [9], we found that binocular measurements with the photoscreener can be made in strabismic children when strabismus can be corrected with the glasses on.

### Refraction changes

Manifest refraction and cycloplegic refraction are the gold standards for lens prescription in children [26]. Therefore, PLUSOPTIX_A09 measurements over spectacles were compared to the refraction measurements obtained during ophthalmological examination. We observed that PLUSOPTIX_A09 measurements over spectacles of the SP and SE strongly correlated with the changes in SP and SE detected during examination.

Moreover, this study established a linear equation to predict refraction changes from Plusoptix measurements over spectacles in children with myopia and mixed astigmatism. Currently, refraction methods only calculate the refraction the child needs to achieve BCVA and do not directly estimate the difference from previous glass prescription [26]. Our results suggest that performing PLUSOPTIX_A09 in a child with their current glasses before subjective refraction could be very helpful, as it would be an easy and rapid procedure to indicate the practitioner if the child needs to update their glasses prescription while also quantifying the adjustment in refraction the child requires.

### Children’s self-perception of VA changes

Assessing children’s self-perception of visual function during anamnesis is highly recommended [27]. Good association between self-report and presence of VA disorders in adolescents has been reported in other studies [28]. Our results suggest that although asking a 10–18-year-old child if they think they still have good vision with glasses is a reliable and easy way of screening a decrease in VA, PLUSOPTIX_A09 over spectacles is a better predictor of VA changes and should be preferred. Furthermore, we found that self-perception is not reliable in younger children (4–9 years old). We believe that self-perception is not reliable in younger children because self-perception is a subjective test, so it may be easier for older children to realize a decrease in VA. Moreover, self-report of vision impairment is limited in children with autism and intellectual disabilities [29]. In this population, Plusoptix seems to be a valuable alternative [30].

### Limitations and future research

Regarding a possible selection bias, we believe that if it exists, it was of minor relevance, as almost all children surveyed during this period had measurements taken with Plusoptix. Nevertheless, it is not possible to completely rule out the presence of selection bias. When performing PLUSOPTIX_A09 over spectacles, glasses manufacturing, cleaning conditions and maintenance state can also influence PLUSOPTIX_A09 readings and interfere with the detection of RE progression. Moreover, although PLUSOPTIX_A09 nonmeasurable results have proven to be important in amblyopia screening [20], these cases were excluded from the study, since only 2 have had out-of-range results, limiting its inclusion in the statistical analysis. During ophthalmological evaluation, the examiner was not blinded for PLUSOPTIX_A09 over spectacles readings, which could have biased VA evaluation. Cycloplegia was performed in 72.0% of the patients with decreased VA. In the other 28.0%, glasses were prescribed according to subjective refraction findings (of those, only 7 patients required an adjustment in refraction greater than 0.50D). Subjective refraction is less sensitive in measuring RE, as it overestimates myopia [31]. In this study, PLUSOPTIX_A09 measurements over spectacles also overestimated myopia progression: A decrease in − 0.10D in the SP and SE measured with the PLUSOPTIX_A09 over spectacles only corresponded to a decrease in refraction of − 0.04D and − 0.05D, respectively. Therefore, not performing cycloplegia in every myopic child with decreased VA may have favored PLUSOPTIX_A09 measurements in this study. Finally, when assessing self-perception of VA changes, the question’s formulation may have biased the child to give a positive answer.

Future researches with children over glasses using other photoscreeners available in the market [32] should be done, as finding more effective devices would increase worldwide accessibility to photoscreening over spectacles.

Furthermore, this study concludes that a decrease in Plusoptix_A09 measurements predicts myopic changes in children wearing glasses. The changes we assessed were: a change in refraction and a change in visual acuity. Both are a sign of myopia progression. However, myopia progression can also be assessed by axial length that was not used in this study. Maybe in future studies axial length could be included.

In conclusion, this study unveiled new promising features for the Plusoptix, a worldwide available photoscreener. When PLUSOPTIX_A09 is performed in children with their glasses on, it can rapidly predict myopia progression, an increasing epidemic in ophthalmology. Values of SP < − 0.06D or SE < − 0.22D indicate VA has decreased, with high sensitivity and specificity. Furthermore, Plusoptix is more reliable than children’s self-perception of VA changes, in 4- to 18-year-old myopic children already wearing glasses. In a matter of seconds, both the ophthalmologist and the primary care clinician can quickly assess whether a patient's myopia has progressed, a method far more precise than asking the child if their vision has diminished.

This study strongly recommends Plusoptix to be routinely performed as an initial assessment in children with myopia, either in primary care or in ophthalmology practice.
